# Dietary Behaviors, Digestive Symptoms, and Neurovegetative Features in Disorders of Gut–Brain Interaction: A Cross-Sectional Clinical Study

**DOI:** 10.3390/nu18071023

**Published:** 2026-03-24

**Authors:** Lavinia Cristina Moleriu, Raluca Lupusoru, Călin Muntean, Teodora Piroș, Alina Popescu, Roxana Sirli, Camelia Nica, Daliborca Cristina Vlad, Dora Mihaela Cîmpian, Diana Mihaela Corodan Comiati, Andrei Luca Dumitrașcu, Victor Dumitrașcu

**Affiliations:** 1 Department III, Functional Science, Discipline of Medical Informatics and Biostatistics, “Victor Babes” University of Medicine and Pharmacy, 300041 Timisoara, Romania; moleriu.lavinia@umft.ro (L.C.M.); raluca.lupusoru@umft.ro (R.L.); teodora.piros@student.umft.ro (T.P.); 2 The Doctoral School of Medicine, “Victor Babes” University of Medicine and Pharmacy, 300041 Timisoara, Romania; andrei.dumitrascu@umft.ro; 3 Center for Modeling Biological Systems and Data Analysis, “Victor Babes” University of Medicine and Pharmacy, 300041 Timisoara, Romania; 4 Gastroenterology and Hepatology Clinic, County Emergency Hospital “Pius Brinzeu”, 300723 Timisoara, Romania; popescu.alina@umft.ro (A.P.); sirli.roxana@umft.ro (R.S.); foncea.camelia@umft.ro (C.N.); 5 Advanced Regional Research Center in Gastroenterology and Hepatology, “Victor Babes” University of Medicine and Pharmacy, 300041 Timisoara, Romania; 6 Department of Internal Medicine II, Division of Gastroenterology and Hepatology, “Victor Babes” University of Medicine and Pharmacy, 300041 Timisoara, Romania; 7 Department IV, Biochemistry and Pharmacology, Discipline of Pharmacology, “Victor Babes” University of Medicine and Pharmacy, 300041 Timisoara, Romania; dumitrascu.victor@umft.ro; 8 Department of Ethics and Social Sciences, George Emil Palade University of Medicine, Pharmacy, Science and Technology of Târgu Mureş, 540139 Târgu Mureş, Romania; dora.cimpian@umfst.ro; 9 Psychiatry Clinic 1, County Clinical Hospital Mureș, 540136 Târgu Mureș, Romania; corodan.mihaela@yahoo.ro

**Keywords:** autonomic nervous system, disorders of gut–brain interaction, eating behavior, functional constipation, gut–brain axis, irritable bowel syndrome

## Abstract

**Background/Objectives**: Disorders of Gut–Brain Interaction (DGBIs), particularly irritable bowel syndrome (IBS), are frequently underdiagnosed in clinical practice, contributing to a substantial hidden burden of disease. This study aimed to quantify this “symptomatic iceberg” by comparing the prevalence of formal IBS diagnoses with a broader symptom-based case definition in a clinical cohort. **Methods**: We conducted a cross-sectional analysis of 194 adult subjects from a gastroenterology clinic in Western Romania. Data on demographics, clinical diagnoses, self-reported symptoms, and eating behaviors were collected. For the case–control analysis, patients with confirmed organic gastrointestinal pathology or incomplete data were excluded. The final analytical sample consisted of 52 patients classified as having a functional DGBI phenotype and 84 asymptomatic controls without organic disease, while 58 were excluded from the analysis. **Results**: While only 4.4% (95% CI: 2.0–9.3%) of the cohort (N = 136) had a formal IBS diagnosis, 47.8% (95% CI: 39.6–56.1%) met criteria for an IBS-compatible symptom cluster, yielding an underdiagnosis ratio of 10.8. Neuro-vegetative symptoms such as sweating (19.1%) and dizziness (11.8%) were highly prevalent. In the case–control analysis, patients with a functional DGBI phenotype had a significantly higher mean BMI compared to controls (28.15 ± 6.49 vs. 24.47 ± 4.60 kg/m^2^; *p* = 0.001). DGBI cases were less likely to report regular snacking behavior (OR = 0.36; 95% CI: 0.18–0.74; *p* = 0.009), suggesting behavioral adaptation. A sensitivity analysis excluding participants with CRP > 10 mg/L (n = 98) confirmed the robustness of these associations, indicating that minor systemic inflammation did not bias the primary findings.

## 1. Introduction

Disorders of Gut–Brain Interaction (DGBIs), with irritable bowel syndrome (IBS) as their archetype, represent a significant global health concern, impacting both patient quality of life and healthcare resources [1]. Defined by the Rome IV criteria as recurrent abdominal pain associated with altered defecation [2]. IBS is a complex and often elusive diagnosis. Recent meta-analyses applying these stringent criteria place the global prevalence of IBS between 4% and 17%, depending on the population and specific criteria used, yet these figures are widely believed to represent only the tip of a much larger “symptomatic iceberg” [3,4]. Diagnostic delays are common, with systematic reviews showing that many patients wait more than a year for a diagnosis, a period marked by persistent symptoms and escalating anxiety. Such delays may contribute to disease progression, increased healthcare utilization, and a reduced quality of life before appropriate treatment is initiated [5]. This diagnostic gap underscores a critical disconnect between the formal definition of the disease and the clinical reality experienced by patients [6]. A phenomenon conceptualized as the “iceberg” of DGBIs where the visible tip of formal diagnoses belies a vast submerged mass of symptomatic individuals [7,8].

The clinical manifestations of DGBIs extend far beyond the gut. Patients frequently report extra-intestinal symptoms such as fatigue, headaches, and autonomic disturbances including palpitations, sweating, and dizziness, palpitations, sweating, and dizziness [9]. These symptoms are manifestations of a dysregulated gut–brain axis, the intricate bidirectional communication network linking the central and enteric nervous systems [10,11]. This axis operates through multiple pathways including vagal afferents, the hypothalamic–pituitary–adrenal (HPA) axis, and the enteric microbiota [12,13]. Emerging research in 2025 continues to highlight the role of reduced vagal tone and autonomic imbalance in the pathophysiology of functional GI disorders, providing a biological basis for these systemic complaints [10,14,15,16]. Studies utilizing heart rate variability (HRV) as a proxy for autonomic function have consistently demonstrated reduced parasympathetic (vagal) activity in IBS patients compared to healthy controls [17]. Such symptoms can mimic primary cardiac or neurological conditions, further complicate the diagnostic process and contributing to the underdiagnosis of the underlying DGBI [18]. The recent post-pandemic surge in DGBIs further emphasizes the impact of stress and environmental factors on the gut–brain axis [19,20].

### The Gut–Brain Axis: Mechanisms and Clinical Implications for Patient Comfort

The gut–brain axis (GBA) represents a complex, bidirectional communication system that integrates neural, hormonal, and immunological signaling pathways between the gastrointestinal tract and the central nervous system [12,13]. This axis is fundamental to understanding why patients with DGBIs often present with symptoms that transcend the gastrointestinal system and why their overall comfort and quality of life are so profoundly affected [20,21]. At the core of this communication lies the vagus nerve, which serves as the primary conduit for afferent signals from the gut to the brain and efferent signals mediating parasympathetic control of digestion [10]. Reduced vagal tone, a hallmark of autonomic dysfunction in DGBI patients, has been linked to impaired gut motility, increased visceral sensitivity, and a heightened perception of pain, all factors that directly diminish patient comfort [16,22].

The concept of “patient comfort” in DGBIs extends beyond the mere absence of pain. It encompasses the patient’s ability to engage in daily activities without the constant preoccupation with gastrointestinal symptoms, the freedom from anxiety about symptom triggers, and the restoration of a sense of control over one’s own body [21,23]. The gut–brain axis directly modulates these aspects through its influence on visceral perception and central pain processing [22]. Visceral hypersensitivity, a core feature of IBS, is not simply a peripheral phenomenon but involves altered processing in brain regions associated with emotion and cognition, including the anterior cingulate cortex and the insula [12,24]. Consequently, interventions that target the gut–brain axis, such as neuromodulators, cognitive-behavioral therapy, and mindful eating practices, have shown efficacy not only in reducing symptom severity but also in improving overall patient well-being and comfort [23,25,26].

Furthermore, the gut microbiota plays an increasingly recognized role in modulating the gut–brain axis [13,27,28,29,30]. Dysbiosis, characterized by alterations in the composition and function of the intestinal microbiome, has been associated with both gastrointestinal symptoms and extra-intestinal manifestations such as anxiety and depression—conditions that significantly impair patient comfort [28,31]. Recent research has identified specific microbial signatures associated with IBS severity [28] opening avenues for targeted probiotic or dietary interventions that could restore microbial balance and, by extension, improve gut–brain communication and patient comfort. In line with these approaches, integrated medical and digital strategies, such as the NutriMonitCare system, have demonstrated the potential to monitor and optimize post-intervention dietary and behavioral factors, supporting microbiome health and enhancing overall patient outcomes [31,32]. Lifestyle and behavior, particularly eating behaviors, may modulate the gut–brain axis and symptom severity. Chrononutrition, the study of how meal timing influences physiological processes, suggests that irregular eating schedules or fast, distracted eating can disrupt circadian rhythms of the gut microbiome, impair motility, and promote low-grade intestinal inflammation. Complementary dietary strategies, including probiotic supplementation combined with nutrients such as L-glutamine and biotin, have been shown to positively influence microbiome composition, body composition, and quality of life in patients with gastrointestinal disorders, highlighting the potential for targeted nutritional interventions to support gut–brain communication and symptom management [33,34,35,36,37]. The gut microbiome itself exhibits diurnal oscillations that are entrained by meal timing [35] and disruption of these rhythms has been linked to metabolic dysfunction and altered gut motility [38,39,40]. Similarly, behaviors such as “fast eating” or “distracted eating” can override natural satiety signals and place undue stress on the digestive system, exacerbating symptoms like bloating and discomfort, a link supported by systematic reviews [41]. Mindful eating practices, in contrast, have been shown to decrease stress and improve digestive function in IBS patients [25,26]. Dietary patterns are also strongly associated with IBS prevalence, as shown in large international studies [42] with specific interventions such as the low FODMAP diet demonstrating efficacy in symptom management [43,44,45,46]. Emerging evidence also suggests that targeting oxidative stress through dietary or antioxidant strategies may further modulate gastrointestinal inflammation and symptom severity, supporting the rationale for precision nutrition approaches in functional bowel disorders [47]. This study aims to quantify the symptomatic iceberg in a clinical cohort from Western Romania. By applying a broader, symptom-based case definition grounded in the principles of the Rome IV criteria [48], we seek to: (1) measure the discrepancy between formal IBS diagnoses and the prevalence of IBS-compatible symptom clusters; (2) characterize the full spectrum of transit-related versus neuro-vegetative symptoms; (3) investigate the association between the functional DGBI phenotype, BMI, and eating behaviors using a rigorous case–control methodology; and (4) discuss the implications of these findings for patient comfort and clinical management. We hypothesize that a significant, clinically relevant population with DGBIs remains undiagnosed, presenting with a complex phenotype where autonomic symptoms are prominent, metabolic factors play a key role, and adaptive coping behaviors reflect an attempt to minimize discomfort.

## 2. Materials and Methods

### 2.1. Participant Selection and Case–Control Derivation

The study was conducted in full compliance with the ethical principles outlined in the Declaration of Helsinki and adhered to the standards of good biomedical research practice. The study protocol received approval from the Institutional Ethics Committee of the Victor Babeș University of Medicine and Pharmacy Timișoara (Approval No. 95/04.10.2021, 4 October 2021).

Of the 194 adult subjects initially included in the dataset, all were screened for eligibility in case–control analysis.

**Inclusion criteria**.

Adult patients (≥18 years) attending the Gastroenterology and Hepatology Clinic during the study period were eligible for inclusion if they met the following criteria:

(1) availability of complete demographic and clinical records;
(2) documented gastrointestinal symptom assessment recorded during routine clinical evaluation;
(3) availability of anthropometric data sufficient to calculate body mass index (BMI);
(4) completion of the dietary behavior questionnaire used for the study.

Consecutive patients meeting these criteria were included to reflect a real-world clinical population.

**Exclusion criteria**.

Participants were excluded from the case–control analysis if any of the following conditions were present:

(1) confirmed organic gastrointestinal disease, including inflammatory bowel disease, gastrointestinal malignancy, peptic ulcer disease, or advanced hepatobiliary pathology documented in medical records;
(2) incomplete clinical, laboratory, or questionnaire data preventing phenotype classification;
(3) overlapping or indeterminate symptom patterns that did not allow clear categorization as either functional DGBI phenotype or asymptomatic control;
(4) acute medical conditions or laboratory abnormalities suggesting active systemic or inflammatory disease requiring further diagnostic evaluation.
(5) extreme laboratory abnormalities suggestive of acute organic or systemic pathology requiring further diagnostic clarification.

**Figure 1:**
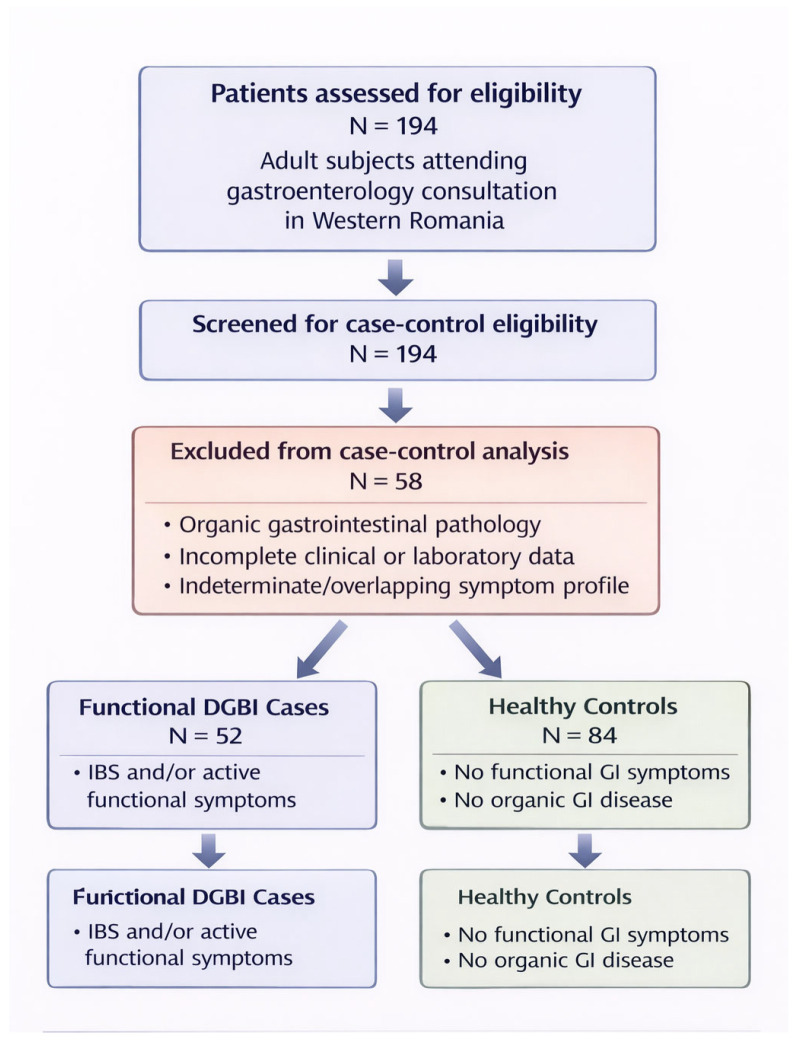
STROBE Flow Diagram of Participant Selection and Case–Control Derivation (N = 194).

Following this screening process, 58 individuals were excluded from the case–control comparison. The final analytical sample consisted of 52 patients classified as having a functional DGBI phenotype (cases) and 84 subjects without functional gastrointestinal symptoms or organic disease (controls). A STROBE-compliant participant flow diagram is presented in Figure 1.

Cases were defined as patients presenting active functional gastrointestinal symptoms compatible with Disorders of Gut–Brain Interaction (DGBIs), operationalized using symptom patterns aligned with Rome IV concepts, in the absence of documented organic gastrointestinal pathology.

Controls consisted of subjects attending the same clinical setting who did not report functional gastrointestinal symptoms and had no evidence of organic gastrointestinal disease based on available clinical evaluation and medical records.

Both cases and controls were derived from the same source population to minimize selection bias and ensure comparability between groups.

This pragmatic design was chosen to balance ecological validity with analytical rigor in a real-world clinical environment.

The study population represents a real-world clinical cohort. While all patients met the Rome IV criteria for DGBI and underwent investigations to rule out organic gastrointestinal pathology, minor systemic biochemical variations were documented, reflecting the comorbid landscape of a typical clinical population.

### 2.2. Data Extraction and Variable Definition

The initial cohort (N = 194) represents the full clinical population assessed during routine care, while inferential statistical analyses were restricted to the rigorously defined case–control subset to improve internal validity.

**Official IBS Diagnosis**: Patients with a recorded diagnosis of “irritable bowel syndrome” or its equivalents.

The IBS-compatible symptom cluster was operationally defined based on symptom patterns consistent with Rome IV concepts but adapted to the structure of our questionnaire dataset. Participants were classified as having an IBS-compatible symptom cluster when they fulfilled the following criteria:

1. Presence of recurrent abdominal discomfort or bloating
2. At least one associated bowel habit alteration (constipation and/or diarrhea)
3. Absence of diagnosed organic gastrointestinal disease according to medical records

**Symptom Identification**: Any variables for transit symptoms (constipation, diarrhea, bloating, abdominal pain, nausea, early satiety) and neuro-vegetative symptoms (sweating, palpitations, dizziness, exhaustion). Autonomic dysregulation was operationalized through the presence of these neuro-vegetative symptoms.

**Food Intolerances**: patients with a diagnosis of lactose intolerance, gluten intolerance, or celiac disease.

**DGBI Phenotype**: To enable a robust case–control analysis, an expanded case definition was created. Cases were defined as patients with IBS, and active functional symptoms while excluding those with organic gastrointestinal pathology (e.g., gastritis, ulcers, Crohn’s disease, Ulcerative rectocolitis). This approach yielded N = 52 cases. Controls were defined as subjects free from both functional symptoms and organic disease (N = 84), creating a “clean” comparison group.

**Eating Behaviors**: Based on the eating habits and meal distribution, variables were created for “fast eater”-self-reported habitual meal duration consistently < 10 min, “distracted eater”, “irregular meals”, and “snacker”.

**Biometric Data**: Body Mass Index (BMI) was calculated from height and weight. Laboratory values for C-reactive protein (CRP), leukocytes, glycemia, HDL cholesterol, LDL Cholesterol, AST, ALT, total bilirubin, Alkaline phosphatase, creatinine, urea, sodium, potassium, GGT—gamma-glutamyl transferase, total cholesterol and triglycerides were also extracted.

### 2.3. Statistical Analysis

Statistical analysis was performed using R (v. 4.3.2; R Foundation for Statistical Computing, Vienna, Austria) and JASP (v. 0.18.3; JASP Team, University of Amsterdam, The Netherlands). Descriptive statistics for continuous variables were reported as mean ± standard deviation (SD) and 95% confidence intervals (CI). The normality of data distribution was assessed using the Shapiro–Wilk test. Prevalence of categorical variables was calculated with 95% Wilson score confidence intervals, which provide robust coverage for small proportions.

For the case–control analysis of eating behaviors, the Chi-square (χ^2^) test was employed, with Odds Ratios (OR) and their corresponding 95% confidence intervals calculated to quantify the strength of association. When expected cell frequencies were below 5, Fisher’s exact test was applied. Comparison of continuous variables (BMI) between groups was performed using Welch’s *t*-test, which does not assume homogeneity of variances. Effect sizes were reported using Cohen’s d for continuous variables and Cramér’s V for categorical associations.

Correlation analyses were conducted using Spearman’s rank correlation coefficient (ρ) for non-normally distributed variables. All statistical tests were two-tailed, and a *p*-value < 0.05 was considered statistically significant and a sensitivity analysis was performed to ensure data robustness. Data visualization was generated using the ggplot2 package in R.

## 3. Results

### 3.1. Descriptive Characteristics of the Cohort

The study cohort (N = 136) had a mean age of 68.70 ± 12.94 years and a mean BMI of 25.36 ± 5.36 kg/m^2^, indicating a population that is, on average, overweight. Detailed descriptive statistics, including a comprehensive biochemical profile, are presented in Table 1.

### 3.2. Spectrum of Symptoms and the Primacy of the Gut–Brain Axis

**Figure 2:**
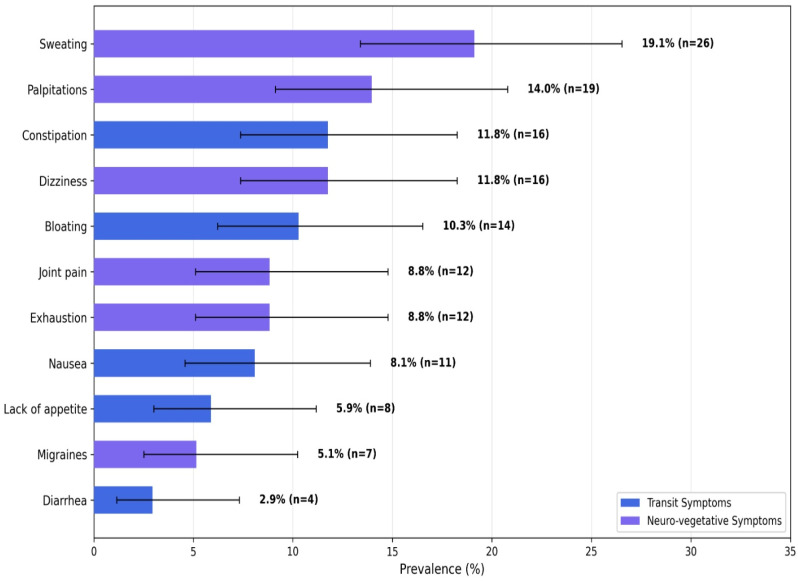
Prevalence of Transit and Neuro-vegetative Symptoms with 95% Wilson Confidence Intervals (N = 136). This horizontal bar chart displays the prevalence of the most frequently reported symptoms, categorized as either transit-related (blue) or neuro-vegetative (purple). Error bars represent the 95% Wilson score confidence intervals. Symptoms are sorted in descending order of prevalence.

Contrary to a gut-centric view, neuro-vegetative symptoms were more prevalent than any single transit disorder (Figure 2). Sweating was the most frequently reported problem (19.1%; 95% CI: 13.4–26.5%), followed by palpitations (14.0%; 95% CI: 9.1–20.8%) and dizziness (11.8%; 95% CI: 7.4–18.3%). Among transit symptoms, bloating was most common 11.8% (95% CI: 7.4–18.3%), followed by constipation 10.3% (95% CI: 6.2–16.5%). This pattern aligns with research demonstrating that autonomic dysfunction is a core feature of DGBIs, often overshadowing the classical bowel symptoms [10,14,15,16].

### 3.3. The Symptomatic Iceberg: Quantifying the Diagnostic Gap

**Figure 3:**
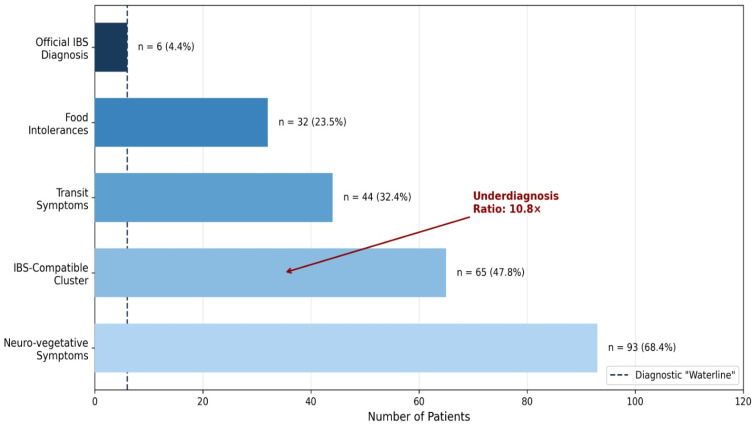
The Symptomatic “Iceberg”: Official Diagnosis vs. Symptom Burden (N = 136). This visualization illustrates the significant discrepancy between the number of patients with a formal IBS diagnosis (the visible “tip” of the iceberg) and the much larger population experiencing a clinically relevant symptom burden (the submerged part). The dashed “diagnostic waterline” highlights the threshold of formal diagnosis. The underdiagnosis ratio of 10.8× is annotated.

The analysis revealed a profound discrepancy between formal diagnoses and symptom prevalence, consistent with the “iceberg” phenomenon described in the literature [7,8,49] (Figure 3). While only 4.4% (n = 6; 95% CI: 2.0–9.3%) of the 136 subjects had an official IBS diagnosis, 47.8% (n = 65; 95% CI: 39.6–56.1%) met the criteria for the broader, clinically relevant IBS-compatible symptom cluster. This yields an underdiagnosis ratio of 10.8, suggesting that for every formally diagnosed patient, nearly eleven others experience a comparable symptom burden without a formal label.

### 3.4. Lifestyle Factors: Eating Behaviors and Transit Patterns

**Figure 4:**
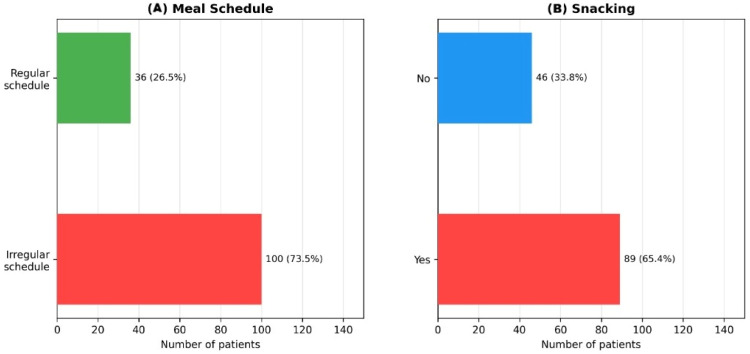
Distribution of Eating Behaviors in the Study Population (N = 136). This panel of three bar charts details the prevalence of key maladaptive eating behaviors. (**A**) illustrates the high prevalence of irregular meal schedules. (**B**) quantifies the proportion of subjects who engage in regular snacking.

Maladaptive eating patterns were nearly ubiquitous (Figure 4). Distracted eating, defined as food intake while simultaneously engaging in other activities, was reported by 50.0% of participants (n = 68). A further 27.2% (n = 37) reported eating in a hurry, while only 22.1% (n = 30) endorsed unhurried eating habits. Irregular meal schedules were observed in 73.5% of subjects (n = 100), and 65.4% (n = 89) reported regular snacking behavior. To ensure conceptual clarity, eating behaviors were analyzed as distinct constructs:

eating speed (fast eating)

attentional eating behavior (distracted eating)

meal timing regularity

snacking behavior

These variables represent complementary but independent behavioral domains rather than components of a single construct.

**Figure 5:**
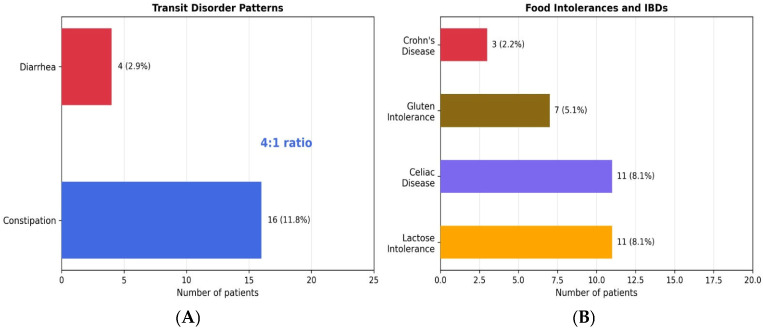
Transit Disorder Patterns and Food Intolerances (N = 136). This panel provides a detailed view of specific transit issues. (**A**) compares the number of patients with constipation versus diarrhea, highlighting the 4:1 predominance of a slow-transit phenotype. (**B**) shows the prevalence of diagnosed food intolerances (lactose, gluten) and related inflammatory bowel diseases (Celiac, Crohn’s), which can overlap with or mimic DGBI symptoms.

Regarding transit patterns (Figure 5), constipation (11.8%, n = 16) was reported approximately four times more frequently than diarrhea (2.9%, n = 4), indicating a predominance of a slow-transit phenotype within the study population. Among diagnosed food-related conditions, lactose intolerance and celiac disease were equally prevalent (8.1% each, n = 11), followed by gluten intolerance (5.1%, n = 7) and Crohn’s disease (2.2%, n = 3).

### 3.5. Case–Control Analysis: Eating Behaviors and BMI in DGBI

To move beyond correlational analysis, a rigorous case–control design was employed, comparing 52 patients with a defined functional DGBI phenotype (cases) against 84 healthy controls without functional or organic GI disease. The results are presented in Table 2.

**Table 2:** (**A**). Eating Behaviors and the Risk of DGBI (Case–Control Analysis). *p*-values were calculated using the chi-square (χ^2^) test for the comparison of proportions between groups. (**B**). Metabolic Profile (BMI) and Low-Grade Inflammation. *p*-values were calculated using Welch’s *t*-test for the comparison of group means.

(**A**)				
**Eating Behavior** **(Risk Factor)**	**DGBI Cases** **(N = 52)**	**Asymptomatic Controls** **(N = 84)**	**Odds Ratio** **(95% CI)**	***p*-Value** **(χ^2^ Test)**
Fast Eating	9 (17.3%)	23 (27.4%)	0.56 (0.23–1.32)	0.255
Distracted Eating	28 (53.8%)	42 (50.0%)	1.17 (0.58–2.33)	0.795
Irregular Meals	34 (65.4%)	57 (67.9%)	0.89 (0.43–1.86)	0.912
Snacking	24 (46.2%)	59 (70.2%)	0.36 (0.18–0.74)	0.009
(**B**)				
**Metabolic Indicator**	**DGBI Cases** **(Mean ± SD)**	**Asymptomatic Controls** **(Mean ± SD)**	**Mean Difference**	***p*-Value** **(Welch’s *t*-Test)**
BMI (kg/m^2^)	28.15 ± 6.49	24.47 ± 4.60	+3.68 units	0.001

Note: Cases include patients with IBS and active functional symptoms (including constipation), excluding organic pathology.

After being adjusted for sex and age, BMI remains significant, while snacking is low in significance (Table 3).

While doing the multiple correction comparison, snacking becomes borderline, while BMI remains robust. Regarding size effects BMI Cohen’s d was 0.71 and Snacking Cramer’s V was 0.18 (Table 4).

The most striking finding from the case–control analysis is the significant association between the DGBI phenotype and both BMI and snacking behavior. Patients with functional DGBIs have, on average, a BMI in the overweight-to-obese range (28.15 kg/m^2^) compared to the asymptomatic control group (24.47 kg/m^2^). This highly significant difference (*p* = 0.001) suggests an intrinsic link between excess weight—potentially mediated by visceral inflammation or obesity-associated dysbiosis—and the generation of functional digestive symptoms [50,51,52]. Conversely, the paradoxical finding that DGBI cases are significantly less likely to snack (OR = 0.36) suggests possible adaptive avoidance behavior, consistent with the hypothesis that patients may learn through negative conditioning that frequent eating triggers symptoms (e.g., bloating, exaggerated gastrocolic reflex), leading them to adopt a more restrictive eating pattern [53]. This restrictive behavior, while understandable, may paradoxically impact patient comfort by limiting dietary variety and contributing to nutritional deficiencies or further gut dysbiosis.

**Sensitivity Analysis.** To evaluate the potential impact of systemic inflammation on the study outcomes, a sensitivity analysis was performed by excluding participants with CRP levels > 10 mg/L (n = 98 remaining in the filtered subgroup). The Chi-square analysis conducted on this filtered subgroup yielded results consistent with the primary analysis of the full cohort (N = 136). Specifically, the associations remained statistically stable for neuro-vegetative features (*p* = 0.37 vs. *p* = 0.41), transit symptoms (*p* = 0.56 vs. *p* = 0.91), IBS confirmed diagnosis (*p* = 0.08 vs. *p* = 0.35), and IBS compatible cluster (*p* = 0.48 vs. *p* = 0.35). These findings indicate that the inclusion of patients with minor CRP elevations did not bias the overall observations or the directions of the identified associations, supporting the robustness of the data within the DGBI population.

## 4. Discussion

This study provides quantitative clinical evidence supporting the presence of a substantial gap between formally diagnosed IBS and symptom-defined functional gastrointestinal burden within a clinical population.

The findings are consistent with the concept of a “symptomatic iceberg,” suggesting that a considerable proportion of patients experience clinically relevant symptoms without receiving a formal diagnosis [6]. Our data suggest that a large population remains in a diagnostic limbo, burdened by symptoms that significantly impact their quality of life but do not fit neatly into existing diagnostic silos.

The predominance of neuro-vegetative symptoms over classic digestive complaints is a critical finding, reinforcing the centrality of the gut–brain axis in the pathophysiology of DGBIs. The high rates of sweating, dizziness, and palpitations are clinical manifestations of autonomic nervous system dysregulation [14,15,54]. Such symptom patterns have been previously associated with altered gut–brain signaling pathways and may reflect interactions between autonomic regulation and gastrointestinal function described in prior studies [18]. The 2025 Seoul Consensus on IBS management emphasizes the need to recognize these extra-intestinal manifestations as integral to the disorder, rather than as separate comorbidities [55].

The constipation-dominant phenotype (4:1 ratio) observed in our cohort is also significant. This may reflect regional dietary patterns or the unintended consequences of self-imposed restrictive diets [56]. Patients with perceived food intolerances often eliminate entire food groups (e.g., dairy, wheat), which can inadvertently reduce fibre intake and worsen constipation, a phenomenon acknowledged in recent WGO guidelines on the management of constipation [57,58,59]. The limitations of the Rome IV criteria in determining the severity of functional constipation in adults further complicate treatment choices [60]. Although chrono-nutrition frameworks propose links between meal timing and gastrointestinal regulation, circadian or microbiome-related mechanisms were not directly assessed in this study. Therefore, these concepts are discussed only as potential explanatory models requiring future investigation.

Notably, no direct statistical association was identified between individual eating behaviors and specific symptoms. This finding suggests that single behavioral variables may not independently explain symptom variability within a heterogeneous clinical population. The observed high prevalence of these behaviors should therefore be interpreted cautiously and viewed as hypothesis-generating rather than explanatory. The emerging science of chrononutrition suggests that such chaotic eating disrupts the circadian rhythms of the gut microbiome, potentially leading to increased intestinal permeability and inflammatory activation [33,34,61]. Furthermore, emerging evidence suggests that modulating oxidative stress through dietary strategies may play a role in regulating intestinal homeostasis. While these mechanisms have been extensively studied in acute inflammatory conditions such as pancreatitis [47], they provide a valuable theoretical framework for precision nutrition approaches in functional disorders as well. The overall cohort displayed marked variability in biochemical parameters, including elevated inflammatory markers (CRP mean 9.28 ± 0.60 mg/L; median 5 mg/L), reflecting the heterogeneity of a real-world gastroenterology population rather than a pre-screened functional cohort. Importantly, inferential analyses were restricted to the rigorously defined case–control subset after exclusion of patients with clear organic pathology. Finally, the finding of a significantly higher BMI in the DGBI group compared to healthy controls (28.15 vs. 24.47 kg/m^2^, *p* = 0.001) is compelling. This suggests that an elevated BMI is a prominent characteristic of the symptomatic DGBI phenotype in our cohort. This link may be multifactorial, considering that increased adiposity is associated with a state of chronic systemic inflammatory activation (‘metaflammation’), which can significantly impact gut function [50,51]. Additionally, mechanical factors related to visceral fat may alter gut motility. While our current analysis focused on the difference in BMI between cases and controls, the prevalence of overweight status in the symptomatic group suggests that weight management could be an important, yet often overlooked, component of a holistic DGBI management strategy.

In this context, the impact of specific nutritional interventions on body composition and quality of life has also been documented in organic gastrointestinal conditions, such as ulcerative colitis [37]. This suggests that metabolic optimization remains a central pillar in the management of gastrointestinal patients, regardless of whether the disorder is functional or organic in nature.

Alternative explanations should also be considered, including reverse causality (symptoms influencing eating behavior), medication effects, comorbid conditions, and age-related physiological changes, all of which may contribute to the observed associations.

These findings must therefore be interpreted within the constraints of a cross-sectional observational design and a clinically derived cohort. Taken together, the results contribute to an emerging view of DGBIs as multidimensional clinical conditions in which gastrointestinal, behavioral, and systemic factors coexist, rather than operate through single causal pathways.

This study highlights the importance of recognizing sub-diagnostic symptom patterns in clinical practice, which may help reduce diagnostic delay and improve patient-centered management strategies.

Beyond traditional questionnaires, the management of DGBI could benefit from integrated digital platforms for real-time nutritional monitoring. Such systems, recently evaluated in complex metabolic contexts like post-bariatric care [32] could serve as a prototype for enhancing patient adherence to long-term dietary interventions and improving the tracking of functional gastrointestinal symptoms.

## 5. Limitations of the Study

Several limitations should be considered when interpreting these findings. Participants were recruited from a clinical gastroenterology setting, which may introduce selection bias and limit external validity. Compared with community samples, clinic attendees may have higher symptom severity, greater comorbidity burden, and different health-seeking behaviors. Consequently, prevalence estimates (including the underdiagnosis ratio) and associations with lifestyle variables may differ from those in general-population studies. The relatively advanced age of the cohort (mean 68.7 years) may influence symptom perception, comorbidity burden, and medication use, which could affect the presentation of DGBIs. Additionally, the biochemical heterogeneity observed in this real-world clinical population reflects routine clinical variability and may complicate the distinction between functional and organic contributors to gastrointestinal symptoms. Data on eating behaviors and symptoms were obtained through self-reported questionnaires, some of which were not based on fully validated instruments, introducing potential recall and reporting bias.

Although the biochemical profile reflected real-world clinical heterogeneity, we acknowledge the presence of laboratory outliers in the initial cohort. To mitigate this, we performed a rigorous sensitivity analysis by excluding participants with CRP > 10 mg/L, which confirmed that these variations did not bias our primary statistical conclusions. However, laboratory parameters were collected for clinical characterization rather than mechanistic analysis. In addition, potential confounding factors, including comorbidities, medication use, and psychological variables, were not fully controlled, and objective physiological measures (e.g., autonomic testing or dietary quantification) were not performed.

The operational definition of the IBS-compatible symptom cluster did not fully reproduce Rome IV diagnostic criteria, and the cross-sectional design precludes causal inference. Therefore, the observed relationships should be interpreted as associative and hypothesis-generating rather than causal.

Despite these limitations, the study benefits from a clearly defined case–control derivation within a single clinical source population and the use of multivariable modeling to reduce confounding. The findings should be viewed as hypothesis-generating and warrant confirmation in larger, prospectively designed cohorts.

## 6. Conclusions

This study highlights a substantial discrepancy between formally diagnosed IBS and the broader burden of functional gastrointestinal symptoms within an elderly clinical population, supporting the concept of a “symptomatic iceberg” in disorders of gut–brain interaction (DGBIs).

The findings support a more integrative, patient-centered clinical perspective that complements criteria-based diagnostic approaches by encouraging clinicians to consider autonomic symptoms, lifestyle factors, and metabolic context during assessment.

### Future Directions

Future research should prioritize longitudinal designs and the use of standardized, psychometrically validated instruments for assessing dietary behaviors and lifestyle patterns in DGBI populations, enabling more reliable comparisons across studies and clearer identification of clinically relevant mechanisms.

Future studies should replicate these analyses in younger and population-based samples to assess whether the “symptomatic iceberg” magnitude and the behavioral correlates remain consistent across age strata.

## Figures and Tables

**Table 1:** Descriptive Statistics of the Study Population (N = 136).

Variable	Mean ± SD	95% CI	Median (IQR)	Range
Age (years)	68.70 ± 12.94	66.50–70.90	70.00 (69.00–73.00)	30.0–92.0
BMI (kg/m^2^)	25.36 ± 5.36	24.39–26.32	24.41 (23.54–25.48)	20.1–32.2
ALT (U/L)	44.89 ± 9.81	35.81–53.98	24.00 (21.00–29.00)	6.0–192.0
AST (U/L)	52.20 ± 10.86	43.57–60.83	31.50 (28.00–37.90)	9.0–203.0
Total cholesterol (mg/dL)	142.83 ± 48.23	134.29–151.37	131.00 (126.00–141.00)	37.0–309.0
Triglycerides (mg/dL)	113.10 ± 4.07	105.03–121.17	103.00 (92.52–114.47)	44.0–238.0
CRP (mg/L)	9.28 ± 0.60	8.09–10.47	5 (5.00–6.00)	4.0–29.0

Isolated elevations in CRP were clinical outliers; organic GI disease was excluded via gold-standard investigations in all cases. Table 1 presents the descriptive characteristics of the post-screening study cohort (N = 136). Stratified case–control comparisons are reported separately in Table 2.

**Table 3:** Logistic regression of model DGBI~Age + Sex + BMI + Snacking. *p*-values were derived from logistic regression analysis of the model DGBI~Age + Sex + BMI + Snacking, and odds ratios (ORs) with 95% confidence intervals (CIs) are reported.

Variable	OR	95% CI	*p*-Value
Age	0.985	0.957–1.015	0.318
Sex	0.685	0.296–1.583	0.376
BMI	1.129	1.045–1.219	0.002
Snacking	0.461	0.205–1.035	0.061

**Table 4:** Multiple correction comparison *p*-values were obtained for multiple testing using the Bonferroni correction and the Benjamini–Hochberg false discovery rate (FDR) procedure; effect sizes were estimated using Cohen’s *d* for BMI and Cramer’s *V* for snacking.

Test	*p* Value	Bonferroni	FDR (BH)
Fast Eating	0.255	1.000	0.425
Distracted	0.795	1.000	0.912
Irregular Meals	0.912	1.000	0.912
Snacking	0.009	0.045	0.023
BMI	0.001	0.005	0.005

## Data Availability

The data presented in this study are available on request from the corresponding author. The data is not publicly available due to privacy and ethical restrictions.
